# Large-scale Metabolomic Profiling Identifies Novel Biomarkers for Incident Coronary Heart Disease

**DOI:** 10.1371/journal.pgen.1004801

**Published:** 2014-12-11

**Authors:** Andrea Ganna, Samira Salihovic, Johan Sundström, Corey D. Broeckling, Åsa K. Hedman, Patrik K. E. Magnusson, Nancy L. Pedersen, Anders Larsson, Agneta Siegbahn, Mihkel Zilmer, Jessica Prenni, Johan Ärnlöv, Lars Lind, Tove Fall, Erik Ingelsson

**Affiliations:** 1 Department of Medical Epidemiology and Biostatistics, Karolinska Institutet, Stockholm, Sweden; 2 Department of Medical Sciences, Cardiovascular Epidemiology, Uppsala University, Uppsala, Sweden; 3 Proteomics and Metabolomics Facility, Colorado State University, Fort Collins, Colorado, United States of America; 4 Department of Medical Sciences, Molecular Epidemiology and Science for Life Laboratory, Uppsala University, Uppsala, Sweden; 5 Department of Medical Sciences, Biochemial structure and function, Uppsala University, Uppsala, Sweden; 6 Department of Medical Sciences, Coagulation and inflammation science, Uppsala University, Uppsala, Sweden; 7 Department of Biochemistry, Centre of Excellence for Translational Medicine, University of Tartu, Tartu, Estonia; 8 Department of Biochemistry and Molecular Biology, Colorado State University, Fort Collins, Colorado, United States of America; 9 School of Health and Social Studies, Dalarna University, Falun, Sweden; 10 Wellcome Trust Centre for Human Genetics, University of Oxford, Oxford, United Kingdom; Georgia Institute of Technology, United States of America

## Abstract

Analyses of circulating metabolites in large prospective epidemiological studies could lead to improved prediction and better biological understanding of coronary heart disease (CHD). We performed a mass spectrometry-based non-targeted metabolomics study for association with incident CHD events in 1,028 individuals (131 events; 10 y. median follow-up) with validation in 1,670 individuals (282 events; 3.9 y. median follow-up). Four metabolites were replicated and independent of main cardiovascular risk factors [lysophosphatidylcholine 18∶1 (hazard ratio [HR] per standard deviation [SD] increment = 0.77, P-value<0.001), lysophosphatidylcholine 18∶2 (HR = 0.81, P-value<0.001), monoglyceride 18∶2 (MG 18∶2; HR = 1.18, P-value = 0.011) and sphingomyelin 28∶1 (HR = 0.85, P-value = 0.015)]. Together they contributed to moderate improvements in discrimination and re-classification in addition to traditional risk factors (C-statistic: 0.76 *vs.* 0.75; NRI: 9.2%). MG 18∶2 was associated with CHD independently of triglycerides. Lysophosphatidylcholines were negatively associated with body mass index, C-reactive protein and with less evidence of subclinical cardiovascular disease in additional 970 participants; a reverse pattern was observed for MG 18∶2. MG 18∶2 showed an enrichment (P-value = 0.002) of significant associations with CHD-associated SNPs (P-value = 1.2×10^−7^ for association with rs964184 in the *ZNF259*/*APOA5* region) and a weak, but positive causal effect (odds ratio = 1.05 per SD increment in MG 18∶2, P-value = 0.05) on CHD, as suggested by Mendelian randomization analysis. In conclusion, we identified four lipid-related metabolites with evidence for clinical utility, as well as a causal role in CHD development.

## Author Summary

Non-targeted metabolomic profiling of large population-based studies has become feasible only in the past 1–2 years and this hypothesis-free exploration of the metabolome holds a great potential to fuel the discovery of novel biomarkers for coronary heart disease (CHD). Such biomarkers are not only important for risk stratification and treatment decisions, but can also improve understanding of cardiovascular disease pathophysiology to identify new drug targets. In this study, we investigated the metabolic profiles of more than 3,600 individuals from three population-based studies, and discovered four metabolites that are consistently associated with incident CHD. We integrate genetic and metabolomic analysis to delineate the underlying biological mechanisms and evaluate potential causal effects of the novel biomarkers. Specifically, we found one metabolite to be strongly associated with single nucleotides polymorphisms previously reported for association with CHD, and consistent with a potential causal role in CHD development, as suggested by Mendelian randomization analysis.

## Introduction

Advances in high-throughput technologies can fuel discovery of novel biomarkers for early detection and prevention of coronary heart disease (CHD). Metabolomic profiling, or metabolomics, provides a holistic signature of biochemical activities in humans by detecting and quantifying low-weight molecules (<1,500 Da). Integration of genetic information and metabolomics data can generate new hypotheses regarding underlying pathophysiological processes [1]. Moreover, targeted metabolomics studies have identified several associations between metabolites and cardiovascular disease (CVD) risk [2], [3] highlighting the importance of metabolic pathways in the development of atherosclerosis.

The primary aim of our study was to identify novel CHD biomarkers by performing non-targeted metabolomics profiling in 3,668 individuals free of CHD at baseline from three population-based prospective cohort studies. Our secondary aims were to delineate the underlying biological mechanisms and to evaluate clinical utility, as well as potential causal effects for those metabolites showing strong evidence of association. For these purposes, we analyzed associations with measures of oxidative stress, inflammation and subclinical CVD, as well as integrated metabolomics and genetics data.

## Results

**Figure 1:**
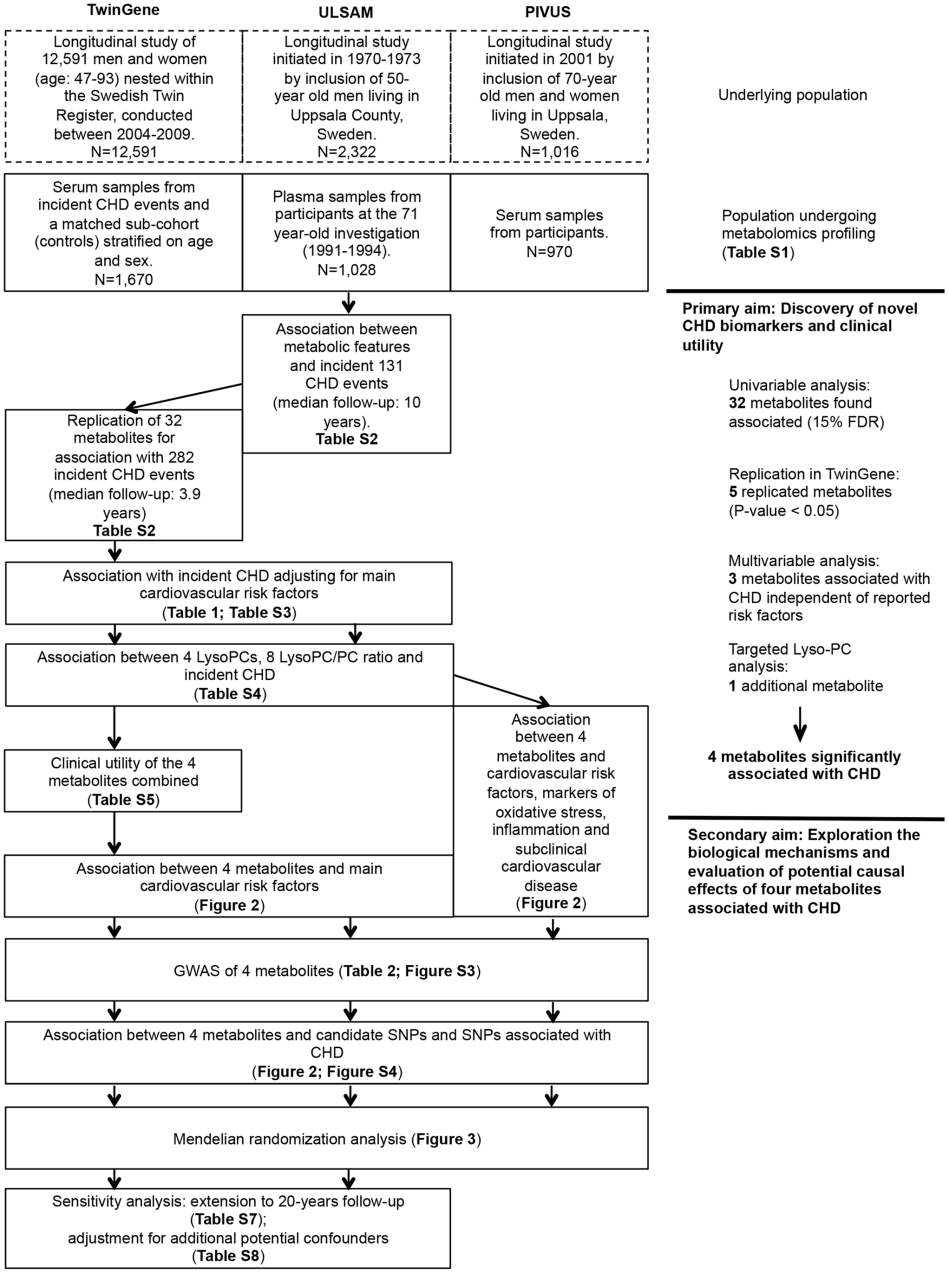
Study flow chart. Overview of the study design and analyses performed.

An overview of the study design is illustrated in **Fig. 1** and baseline characteristics of the three studies are described in **S1 Table**. Participants in ULSAM and PIVUS were all of the same approximate age at baseline (interquartile range, 70.1 to 71.0 years), while TwinGene participants were of younger median age (64.7 years) and with a wider range (interquartile range, 59.2 to 69.9 years).

### Primary aim: Discovery of novel CHD biomarkers and the clinical utility

#### Discovery and validation of metabolic features associated with incident CHD

In the 1,028 ULSAM participants free of CHD events at baseline, we observed 131 CHD events during a median follow-up of 10.0 years. There were 32 unique metabolites associated with CHD incidence at a 15% FDR level (**S2 Table**). Nine metabolites were annotated using our in-house compound library [Metabolomics Standard Initiative (MSI) level 1] and 12 using publically available databases (MSI level 2). We could identify the metabolic class (MSI level 3) for seven metabolites, while four candidate metabolites could not be annotated (MSI level 4).

We sought to replicate these 32 metabolites in the TwinGene study, where 282 incident CHD events were observed during a median follow-up of 3.9 years. Twenty-seven metabolites showed a consistent direction in TwinGene (binomial test P-value <0.001). Five of the metabolites showed significant association (P-value<0.05) and consistent direction [monoglyceride 18∶2 (MG 18∶2), a monosaccharide, lysophosphatidylcholine 18∶2 (Lyso PC 18∶2), a derivative of cinnamic acid and sphingomyelin 28∶1 (SM 28∶1); **Table 1** **and S3 Table**]. Since we detected a significant interaction between LysoPC 18∶2 and age (P-value = 0.03; **S3 Table**), with a stronger protective effect on CHD in individuals older than 70 years (**S1 Figure, Panel A**), we model this interaction in all the following analyses and report estimates also for individuals older than 70 years

**Table 1:** Association between metabolites replicated in the univariable analysis and CHD, adjusting for main cardiovascular risk factors, meta-analysis results from ULSAM and TwinGene (N = 2,698).

Metabolite	Random-effect Meta-analysis*	
	HR (95% CIs)	P-value
**LysoPC 18∶2** †	**0.81 (0.71–0.92)**	**<0.001**
Monosaccharides	1.12 (0.99–1.26)	0.064
**MG 18∶2**	**1.18 (1.04–1.34)**	**0.011**
Cinnamic Acid Derivative	0.89 (0.80–1.00)	0.050
**SM 28∶1**	**0.85 (0.75–0.97)**	**0.015**

* Values are from random effect meta-analysis of Cox proportional hazards analyses for a SD increment of the metabolic feature adjusted by age, sex (only in TwinGene), systolic blood pressure, body mass index, current smoker, antihypertensive treatment, LDL cholesterol, HDL cholesterol, log-triglycerides and diabetes at baseline.

† Significant interaction with age; we modeled an interaction between age and LysoPC 18∶2 and included the estimates for individuals older than 70 in the meta-analysis.

Metabolites in bold showed P-value<0.05 for association with CHD.

Three metabolites (MG 18∶2, Lyso PC 18∶2 and SM 28∶1) remained significantly associated with incident CHD after adjustment for main cardiovascular risk factors and meta-analysis of TwinGene and ULSAM results (**Table 1**); LysoPC 18∶2 (hazard ratio [HR] per standard deviation [SD] increment = 0.81; P-value<0.001) and SM 28∶1 (HR = 0.85; P-value = 0.015) were negatively associated, while MG 18∶2 (HR = 1.18; P-value = 0.011) was positively associated.

Chemical structures of these metabolites were additionally confirmed by targeted tandem mass spectrometry (**S2 Figure**).

#### LysoPCs and their ratios in relation to incident CHD

Since LysoPC 18∶2 was the metabolite with the strongest association with incident CHD in ULSAM and in older participants from TwinGene, we extended our analysis to four additional LysoPC species to evaluate common patterns and pathways. Moreover, since the main mechanism by which LysoPCs are formed is via hydrolysis of phosphatidylcholines (PC) [4], we explored the association between the most abundant LysoPC/PC ratios and incident CHD. There was a strong negative association between LysoPC 18∶1 and incident CHD (HR = 0.77; P-value<0.001) in the two studies combined after adjustment for main cardiovascular risk factors (**S4 Table**). This increased the number of metabolites significantly associated with incident CHD independently of main cardiovascular risk factors to four. Survival curves for each metabolite are reported in **S1 Figure, Panel B**. The ratios between LysoPCs and PC were not significantly associated with CHD (**S4 Table**). LysoPC 18∶1 was highly positively correlated with LysoPC 18∶2 (r^2^ = 0.74, P-value<0.001) and negatively correlated with MG 18∶2 (r^2^ = −0.15, P-value<0.001). Similarly, SM 28∶1 was positively correlated with LysoPC 18∶1 (r^2^ = 0.37, P-value<0.001) and LysoPC 18∶2 (r^2^ = 0.42, P-value<0.001) and negatively correlated with MG 18∶2 (r^2^ = −0.13, P-value<0.001).

#### Clinical utility of four metabolites

Since four metabolites (LysoPC 18∶1, LysoPC 18∶2, MG 18∶2 and SM 28∶1) were associated with CHD after adjustment for main cardiovascular risk factors, we investigated their utility as biomarkers for CHD prediction. When the four metabolites were added to a model comprising the risk factors included in the Framingham Heart Study risk score [5], we observed a modest improvement in C-index (0.759 *vs.* 0.751, P-value = 0.026) and a moderate improvement in the Net Reclassification Index (NRI) (9.9% [1.2; 20.2] for events and −0.7% [−6.0; 0.5] for non-events; **S5 Table**).

### Secondary aim: Exploration of biological mechanisms and evaluation of potential causal effects of four metabolites associated with CHD

#### Association with main cardiovascular risk factors, markers of oxidative stress, inflammation and subclinical CVD

**Figure 2:**
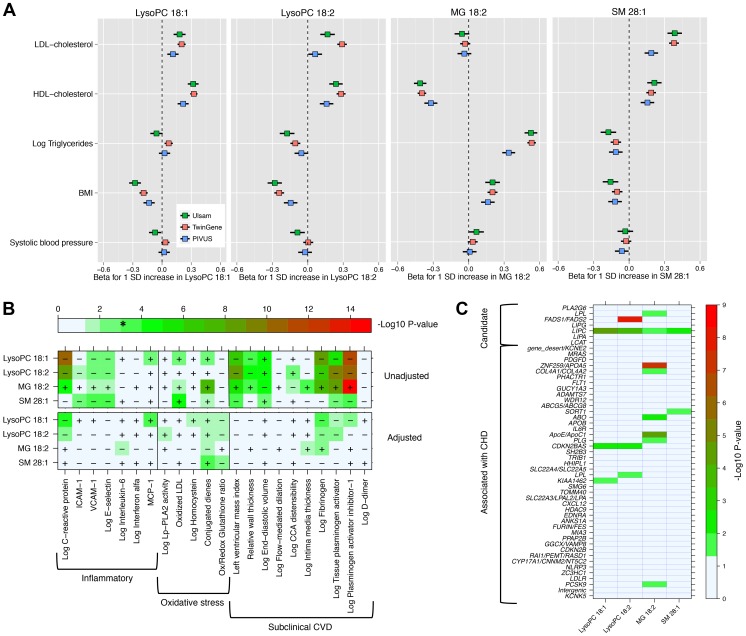
Association between four metabolites and cardiovascular traits and genotypes. **Panel A**: Association with main cardiovascular risk factors in three population-based studies. **Panel B**: Minus log_10_(P-value) for association with markers of inflammation, oxidative stress and subclinical CVD in PIVUS. Sex-adjusted analysis (upper panel) and adjusted by sex, systolic blood pressure, body mass index, current smoker, antihypertensive treatment, LDL-C, HDL-C, log-triglycerides and diabetes at baseline (lower panel). * indicates the alpha threshold after multiple-testing correction. **Panel C**: Minus log_10_(P-value) for association with 51 SNPs previously reported for association with CHD (44 SNPs) or selected from candidate pathways (7 SNPs).

We explored the associations of our four novel metabolites and main cardiovascular risk factors (**Fig. 2** **, Panel A**), as well as with markers of oxidative stress, inflammation and subclinical CVD (**Fig. 2** **, Panel B**). The two LysoPC species showed a similar pattern of association; higher LysoPC levels were associated with higher high-density lipoprotein cholesterol (HDL-C) and low-density lipoprotein cholesterol (LDL-C) levels and lower body mass index (BMI). Similar associations were also observed for SM 28∶1. Monoglyceride 18∶2 was positively associated with triglycerides and BMI levels in all the three studies, while the association with HDL-C levels was in the inverse direction. The correlation between MG 18∶2 and triglycerides (measured in serum using standard methods) was strong (*r* ^2^ range: 0.25–0.53). In TwinGene, when triglycerides and MG 18∶2 were included in the same model adjusting for only age and sex, both showed an independent significant positive association with incident CHD (**S6 Table, panel A**). Further, when they were separately added to models with all main cardiovascular risk factors except triglycerides, MG 18∶2 showed a larger increase in the likelihood ratio (204.6 *vs.* 197.6) and C-statistic (0.755 *vs.* 0.753) compared with triglycerides (**S6 Table, panel B**).

In PIVUS (**Fig. 2** **, Panel B**), we observed the two LysoPC species being associated with lower levels of inflammation markers and less subclinical CVD. We detected a strong inverse association between LysoPC 18∶1 and plasminogen activator inhibitor-1 (PAI-I; P-value = 1.8×10^−12^), C-reactive protein (P-value = 3.1×10^−11^), fibrinogen (P-value = 7.3×10^−9^), and left ventricular mass index (P-value = 2.2×10^−7^). MG 18∶2 was positively associated with several markers of oxidative stress and subclinical CVD: PAI-I (P-value = 1.7×10^−15^), tissue plasminogen activator (P-value = 2.5×10^−9^), fibrogen (P-value = 6.4×10^−8^) and conjugated dienes (P-value = 1.2×10^−8^). After adjustment for main cardiovascular risk factors, most associations were attenuated. However, in these multivariable-adjusted models, LysoPC 18∶1 remained significantly associated with higher levels of monocyte chemotactic protein-1 (MCP-1) and lower levels of C-reactive protein and fibrinogen, even after correction for multiple testing.

#### Genome-wide association studies

We tested for association between ∼7.5M 1000G-imputed single nucleotide polymorphisms (SNPs) and the four metabolites associated with CHD independently of main cardiovascular risk factors (LysoPC 18∶1, LysoPC 18∶2, MG 18∶2 and SM 28∶1) in all 3,620 participants from the three studies with both genetic and metabolomics data (**Table 2**). In analyses of LysoPC 18∶1, we detected a novel association with rs75729820 (P-value = 2.7×10^−8^), close to *C8orf87* on chromosome 8, and a suggestive association signal (rs8141918; P-value = 4.5×10^−7^) close to *A4GALT* on chromosome 22 (**S3 Figure**). We could also confirm a previously reported association between a SNP upstream of the *FADS2* gene and LysoPC 18∶2 [6], and found a suggestive association between rs964184, in the *ZNF259*/*APOA5* region, and MG 18∶2 (P-value = 1.2×10^−7^). The rs964184 variant has been associated with several cardiovascular traits including CHD in previous studies [6], [7]. The SNP rs12878001 near to *SGPP1* was significantly associated with SM 28∶1 and correlates with a SNP (rs17101394; r^2^ = 1) previously reported to be associated with sphingolipid levels [8]. All the SNPs reported in **Table 2** had consistent direction of effect in the three studies.

**Table 2:** GWAS of metabolites associated with CHD; SNPs with P-value<5×10^−7^ and minor allele frequency > 5% are reported.

Metabolite	Chromosome	SNP	Position (build 37)	Nearest Gene	Effect/non- effect allele	Average Allele Frequency	Meta-analysis in the three studies (N = 3,620)*		SNP Context
							OR	P-value	
**LysoPC 18∶1**	8	rs75729820†	94088655	*C8orf87*	T/C	0.95	1.34	2.7E-08	Intergenic
	22	rs8141918	43136583	*A4GALT*	A/G	0.70	1.14	4.5E-07	Intergenic
**LysoPC 18∶2**	11	rs174568	61593816	*FADS2*	T/C	0.35	1.15	8.4E-09	NearGene-5‡
	8	rs2048797	115181070	*CSMD3*	A/T	0.70	1.14	4.4E-07	Intergenic
**MG 18∶2**	11	rs964184	116648917	*ZNF259/APOA5*	G/C	0.13	1.20	1.2E-07	NearGene-3‡
**SM 28∶1**	14	rs12878001	64239629	*SGPP1*	G/T	0.15	1.21	1.2E-08	Intergenic
	1	rs113317091	32925064	*ZBTB8B*	T/C	0.13	1.20	1.9E-07	Intergenic

* Values are from fixed effect meta-analysis.

† Significant heterogeneity across studies (I^2^ = 0.8).

‡ The “Near Gene” region includes the mRNA region of the gene as well as arbitrary regions of 2K nucleotides upstream and 0.5K nucleotides downstream to allow for potential regulatory regions.

#### Association with variants associated with CHD or from candidate pathways

We investigated the association between the four metabolites and 44 established CHD-associated SNPs [9], as well as seven candidate SNPs targeting biologically relevant pathways (**Fig. 2** **, Panel C**; **S1 Text** for SNPs selection procedures). MG 18∶2 showed a significant enrichment of P-values<0.05 for association with CHD-associated SNPs compared to the expected (hypergeometric test P-value = 0.002). This enrichment remained even after adjustment for main cardiovascular risk factors (P-value = 0.02; **S4 Figure**). The other metabolites did not show a significant enrichment of low p-values.

Among candidate SNPs targeting relevant pathways, *LIPC* was associated with all four metabolites, confirming the role of hepatic lipase in regulation of MG and LysoPCs levels. Candidate SNPs in the *FADS1*/*FADS2* region were not strongly associated with MG 18∶2 and LysoPC 18∶1. After adjustment for main cardiovascular risk factors, only the association of *FADS1*/*FADS2* with LysoPC 18∶2 remained genome-wide significant (P-value = 2.2×10^−12^).

#### Mendelian randomization analysis

**Figure 3:**
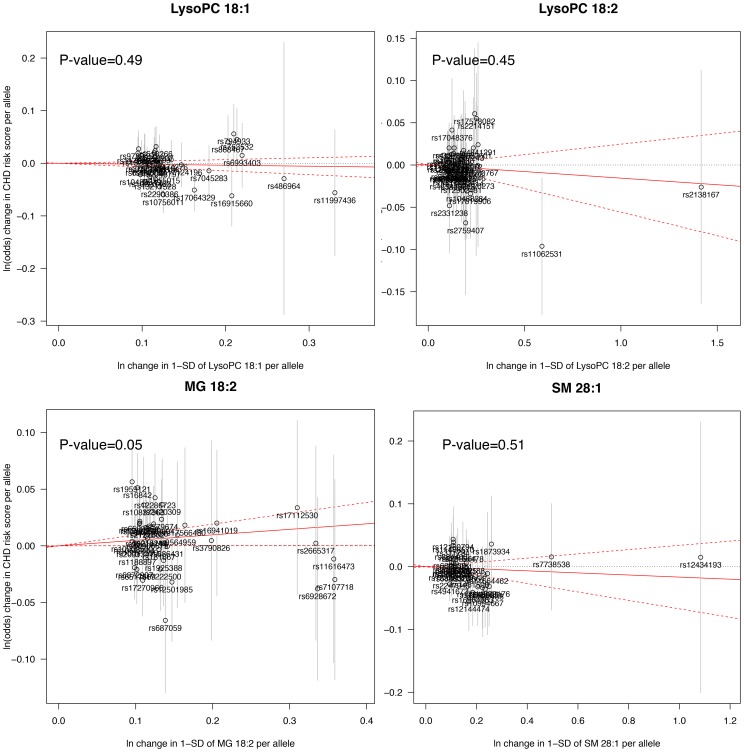
Mendelian randomization analysis. A significant deviation from zero of the estimate of causal effect using all SNPs (solid red line) suggests a causal relationship between the metabolite and CHD.

The Mendelian randomization analysis (**Fig. 3**) suggested a weak, but positive causal effect of MG 18∶2 on CHD risk (odds ratio, 1.05 [95% CI, 1.00–1.10] per SD increment in MG 18∶2; P-value = 0.05) and a lack of causal effect for LysoPC 18∶1, 18∶2 and SM 28∶1.

#### Sensitivity and exploratory analysis

First, we evaluated the association of the four metabolites and incident CHD when the ULSAM follow-up was extended to 20 years, including 198 CHD events (**S7 Table**). The effect sizes were comparable to those observed for the 10-year follow-up. Second, in the multivariable analysis for association with CHD, we separately included two covariates in addition to the main cardiovascular risk factors: C-reactive protein and statin treatment. The associations between the four metabolites and incident CHD were essentially the same (**S8 Table**). Third, we assessed Lp-PLA_2_ activity in 254 older individuals (64 CHD events) from TwinGene. Lp-PLA_2_ is a known marker of atherosclerosis and hydrolyzes PC to produce LysoPCs. By adjusting the analysis for Lp-PLA_2_ we wanted to evaluate if the protective association between LysoPC 18∶1 and incident CHD was confounded or mediated by Lp-PLA_2_. The effect size after adjustment for Lp-PLA_2_ activity in addition to main cardiovascular risk factors was similar as in the main multivariable model (HR = 0.78; P-value = 0.176) arguing against Lp-PLA_2_ being an important confounder or mediator of the association.

## Discussion

### Principal findings

In this study of 3,668 participants from three prospective population-based cohorts, we investigated the association of circulating metabolites measured by liquid chromatography coupled mass spectrometry with incident CHD. In our discovery cohort, 32 metabolites were associated with CHD, of which 84% showed a directionally consistent association with CHD in our validation cohort. In multivariable analyses adjusted for main cardiovascular risk factors, three metabolites remained associated with CHD. In a targeted LysoPC analysis, we detected one additional significant association resulting in a total of four metabolites associated with CHD independently of main cardiovascular risk factors: LysoPC 18∶1, LysoPC 18∶2, MG 18∶2 and SM 28∶1. These biomarkers moderately improved risk reclassification beyond traditional risk factors, when commonly used risk categories were considered. Lysophosphatidylcholines were negatively associated with BMI, markers of inflammations and subclinical cardiovascular disease, while a reverse pattern was observed for MG 18∶2. We found evidences for a causal effect of MG 18∶2 on CHD independently of triglycerides levels. Finally, we uncovered several genome-wide significant SNPs and suggestive signals for association with LysoPCs, some of which have not been previously observed.

### Monoglycerides

We observed a strong positive association between MG 18∶2 and CHD. The majority of circulating monoglycerides are released by the action of lipoprotein lipase and hepatic lipase, which catalyze the hydrolysis of triglycerides to provide non-esterified fatty acids and monoglycerides for tissue utilization [10]. Monoglycerides are further converted into free fatty acids and glycerol by monoglyceride lipase. Within the intestinal wall, monoglycerides are used to resynthesize diglycerides and triglycerides via monoacylglycerol pathway before being transported in lymph to the liver. Several observations suggest an involvement of MG 18∶2 in the pathogenesis of CHD. First, MG 18∶2 is central in the synthesis and breakdown of triglycerides and a causal effect of plasma triglyceride levels on CHD risk have recently been supported by a large Mendelian randomization analysis [11]. Although highly correlated, when both MG 18∶2 and triglycerides were included in the same model, both showed independent significant associations with CHD. Moreover, when separately added to a model with main cardiovascular risk factors, MG 18∶2 was a better predictor of CHD than triglycerides. Second, MG 18∶2 was associated with higher levels of cardiovascular risk factors and markers of subclinical CVD and oxidative stress. Third, Mendelian randomization analysis suggested a weak, but positive causal effect of MG 18∶2 on CHD risk. Several SNPs reported for association with CHD remained associated with MG 18∶2 (in the *PCSK9*, *HHIPL1*, *PLG*, *ApoE*/*ApoC1*, *COL4A1*/*COL4A2* regions, P-values<0.05), even after adjustment for main cardiovascular risk factors.

### LysoPCs

We observed a strong age-dependent association between LysoPC 18∶2, LysoPC 18∶1 and CHD risk, with stronger inverse association in older individuals. These LysoPC species were further characterized to be associated with higher HDL-C and total cholesterol levels, and lower BMI and markers of subclinical CVD. Moreover, they were highly correlated, suggesting shared biological mechanisms. LysoPCs are mostly derived from phosphatidylcholines (PC) and several mechanisms contribute to their formation. A large component of LysoPC in plasma is derived from PC by the glycoprotein lecithin cholesterol acyltransferase (LCAT). Another well-known mechanism of LysoPC production, which mainly takes place in tissues, is via PC hydrolysis by the action of secretory PLA2 family [4]. Although higher levels of LysoPCs have been observed during the oxidative modification of LDL-C that accompanies their conversion to atherogenic particles, it has also been shown that LysoPCs produced by a PLA2-like activity of Paraoxanase 1 contributes to the inhibition of macrophage biosynthesis and that they consequently reduce cellular cholesterol accumulation and atherogenesis [12]. LysoPC are also produced by endothelial lipase and hepatic lipase [13]. Hepatic lipase, which is also involved in triglyceride hydrolysis, is mainly responsible of the production of unsaturated LysoPCs [14], [15]. Although LysoPCs are commonly seen as pro-inflammatory and pro-atherogenic metabolites [16], recent population-based studies have suggested a protective effect of LysoPCs on cardiovascular risk. In a study of type 2 diabetes, LysoPC 18∶2 was found to be inversely associated with incident diabetes and impaired glucose tolerance [17]. Fernandez and colleagues found an inverse association of LysoPC 16∶0 and LysoPC 20∶4 with incident CVD and reduced intima media thickness [18]. More recently, Stegemann and colleagues [19] found an inverse association between several LysoPC species and incident CHD. Our study confirms and extends these previous findings. Using a Mendelian randomization approach, we suggest that the observed association between LysoPCs and incident CHD are likely to not be causal.

### Strengths and limitations

Our study has several strengths. To our knowledge, this is the largest study investigating the metabolome in relation to incident CHD. Mass spectrometry-based metabolomics is extremely sensitive and allows detection of more metabolites than nuclear magnetic resonance-based methods [20]. We validated our findings using an independent population, with a different blood collection method, blood partition (serum instead of plasma) and age range. At the cost of augmented heterogeneity, this approach has the advantage to increase the generalizability of our findings. All three study samples were longitudinal and we have studied incident events decreasing the risk of reverse causation or selection bias as an explanation to our observations. We performed extensive characterization of underlying biological mechanisms, clinical utility, and potential causal effects for those metabolites showing strong evidence of association. We also acknowledge several limitations of our study. First, we used a non-targeted approach, meaning that every ion detected by mass spectrometry was treated as a separate variable, increasing the multiple-testing burden. We have previously shown that this approach does not affect the FDR point estimate, but might increase its variability [21]. However, this method is advantageous because it does not rely on pre-annotation and allows inclusion of unknown metabolites in the analyses (which subsequently can be identified using targeted methods). Moreover, we used a single analytical platform (liquid chromatography-mass spectrometry); the integration of multiple analytical platforms is a way of increasing the number of detectable metabolites. Second, non-targeted metabolomics is subject to co-elution of metabolites, ion suppression and imprecision in metabolites quantification, since each value assigned to the metabolic feature can only be interpreted as mass ion intensity. However, we do not have reason to believe that such biases would systematically affect CHD cases, since our outcome is measured prospectively and metabolomic profiling performed in a blinded fashion. Moreover, each sample has been analyzed in non-consecutive randomized duplicates, which minimize the risk of systematic biases. Third, the use of 15% FDR in the discovery phase is larger than in some other studies, but is justified by the high degree of correlation in the data, due to the existence of multiple metabolic features for a single metabolite. Moreover, metabolites were replicated (P-value<0.05) in an independent study sample. To evaluate if our replication strategy was sufficient to minimize the number of false positives, we estimated the expected false discovery rate in the replication sample (TwinGene) [22]. This was calculated as 0.23% (**S1 Text**), meaning that only 0.23% of metabolites replicating at P<0.05 are expected to be false positives, suggesting that our two-tier approach correctly control the number of false positives.

Finally, as our study samples consist of middle-aged to elderly individuals of Northern European decent, the generalizability to other ethnicities and younger age groups is unknown.

### Conclusions and future directions

In conclusion, in the largest study of the metabolome in relation to incident CHD to date, we identified lysophosphatidylcholines 18∶1, 18∶2, monoglyceride 18∶2 and sphingomyelin 28∶1 as risk factors of coronary heart disease and suggested a causal effect for monoglyceride 18∶2 on CHD. Future experiments should mainly focus on determining the mechanisms by which these metabolites of lipid metabolism might be involved in pathogenesis of coronary heart disease.

## Materials and Methods

### Study samples

We performed metabolomic profiling of blood samples from three studies: TwinGene, ULSAM and PIVUS. An overview of the study design is illustrated in **Fig. 1**, and a detailed description of each study is given in the **S1 Text**.

In brief, TwinGene is a longitudinal sub-study of 12,591 individuals (55% women) from the Swedish Twin Register [23]. For the purpose of metabolomic profiling, we designed a case-cohort of incident CHD events and a matched sub-cohort (controls) stratified on age and sex [24]. In the final analysis we included serum samples from 1,670 unrelated individuals.

The Uppsala Longitudinal Study of Adult Men [25] (ULSAM; http://www2.pubcare.uu.se/ULSAM/) is an ongoing, longitudinal, epidemiologic study of men born between 1920 and 1924 in Uppsala County, Sweden. In the final analysis, we included plasma samples from 1,028 individuals investigated at 70 years of age.

The Prospective Investigation of the Vasculature in Uppsala Seniors [26] (PIVUS; http://www.medsci.uu.se/pivus/) is a population-based study of 70-year old individuals living in Uppsala. In the final analysis, we included serum samples from 970 individuals.

Incident CHD cases were defined as hospitalization or death with a primary diagnosis for acute myocardial infarction or unstable angina. This information was collected by linking the personal identity numbers from TwinGene and ULSAM participants with the Swedish National In-Patient Register and the Cause of Death Register up to the 31^th^ December 2010, which comprise the end of follow-up of the present study.

### Laboratory measurements

Laboratory procedures for metabolomics have been previously described [21], [27] and are detailed in the **S1 Text**. Briefly, metabolomic profiling was performed on Acquity UPLC coupled to a Xevo G2 Q-TOFMS (Waters Corporation, Milford, USA) with an atmospheric electrospray interface operating in positive ion mode. Non-consecutive duplicate sample aliquots of 1 µL were injected onto a Acquity UPLC BEH C8 analytical column. Mass analysis was performed in the full scan mode (m/z 50–1200).

Genotyping arrays used in each study are described in the **S1 Text**. All the samples underwent the same quality control (QC) and imputation of polymorphic 1000 genome CEU SNPs (Phase I, version 3) performed using IMPUTE2.

Methods for measuring the 21 biological markers and imaging features in PIVUS have been previously described [28]–[30].

### Metabolic feature detection and annotation

Raw data were processed using XCMS software [31]. Procedures to perform non-targeted metabolomics in large-population studies have been previously described by our group [21]; the code has been made publically available at https://github.com/andgan/metabolomics_pipeline. Metabolic feature detection, alignment, grouping, imputation and normalization were performed separately for each study (**S1 Text**). Each feature is characterized by a specific mass-to-charge ratio (m/z) and retention time. A single metabolite is normally represented by more than one feature. Indiscriminant (id) MS and idMS/MS spectra were generated for all the significant features [27]. Those with highly similar spectra, strong correlation and similar retention time were deemed to be from the same metabolite. We used the spectra to identify the corresponding metabolite. Four approaches were considered, in agreement with what has been suggested by the Metabolomics Standard Initiative (MSI) [32] and as described in the **S1 Text**.

### Statistical analysis

In ULSAM, we tested the association between each feature and incident CHD using a Cox proportional hazards model adjusted by age at baseline. We restricted our analysis to a 10-year follow-up since most biological markers experience a decreasing association with longer follow-up due to regression dilution bias. To evaluate the proportional hazard assumption we obtained, for each feature, a P-value from the Schoenfeld residual-based test; we did not detect any significant deviation from the proportionality assumption after correcting for multiple testing.

Features that were significantly associated with CHD in ULSAM at 15% false discovery rate (FDR) level were taken forward for replication in TwinGene. In TwinGene, we fitted Cox models adjusted for age and sex, and re-weighted for the inverse of the sampling probability using the Borgan “Estimator II” [24]. Features with P-value<0.05 in TwinGene and showed association with consistent direction were considered as replicated.

In the multivariable analysis, we studied the association between replicated features and CHD adjusting for main cardiovascular risk factors (sex, age, systolic blood pressure, BMI, current smoking, antihypertensive treatment, LDL-C, HDL-C, natural logarithm-transformed triglycerides and prevalent diabetes). Association analyses between metabolic features and markers of oxidative stress, inflammation and subclinical CVD in PIVUS was performed using linear regression adjusted only for age and sex, and for the same cardiovascular risk factors described above.

In TwinGene, reclassification measures (NRI, see **S1 Text** for additional details) were calculated using a 10% and 20% threshold for a 10-year risk of event, as often done in previous literature [33].

The genome-wide association study (GWAS) analyses were performed in PLINK adjusting for age, sex (where feasible) and first three principal components; results were meta-analyzed using fixed effects inverse-variance weighted meta-analysis in METAL. Instrumental variables for the Mendelian randomization analysis were constructed using the GWAS results and tested for association with CHD using the results from the CARDIOoGRAMplusC4D consortium [9]. Criteria for exclusion of pleiotropic SNPs and additional methodological information can be found in the **S1 Text**.

### Ethics statement

All participants gave informed written consent and the Ethics Committees of Karolinska Institutet or Uppsala University approved the respective study protocol.

## Supporting Information

S1 Figure **Panel A**: Hazard Ratio (HR) for association between LysoPC 18∶2 and incident CHD as function of age, modelled using splines. The association between LysoPC 18∶2 and CHD is stronger at older age, starting from around 70-years old. **Panel B**: Survival curves for time-to-CHD for tertiles of each metabolite. We fixed the other covariates so that the curves are representative of a men, 77 years old, smoker, not antihypertensive drugs user and not diabetic with systolic blood pressure = 150, BMI = 26, LDL-C = 2.6 mmol/l, HDL-C = 1.3 mmol/l and triglycerides = 1.7 mmol/l.
(PDF)

S2 Figure Product ion spectra of selected metabolites (upper spectrum) and their corresponding standards (lower spectrum) using a UPLC-QTOFMS operated in ESI positive mode. **Panel A**: Metabolite eluting at 6.37 minutes represented by [M+H]^+^ = 522.356 identified as LysoPC 18∶1. **Panel B**: Metabolite eluting at 5.78 represented by [M+H]^+^ = 520.341 identified as LysoPC 18∶2. **Panel C**: Metabolite eluting at 6.40 represented by [M+H]^+^ = 337.269 identified as MG 18∶2. **Panel D**: Product ion spectra of a SM 28∶1 (C_36_H_73_N_2_O_6_P, M 660.521 Da) and its fragmentation pattern using a UPLC-QTOFMS operated in ESI positive mode where the protonated molecule [M+H]^+^ = 661.526 and the fragment ions at m/z 184.074 and m/z 86.097 represented by the loss of a phosphocholine group and a choline group, respectively.
(PDF)

S3 Figure LocusZoom plots of the two top signals for association with LysoPC 18∶1.
(PDF)

S4 Figure Minus log_10_(P-value) for association between four metabolites and 51 SNPs previously reported for association with CHD (44 SNPs) or selected from candidate pathways (7 SNPs) after adjustment for main cardiovascular risk factors.
(PDF)

S1 Table Baseline descriptive statistics for main cardiovascular risk factors.
(XLSX)

S2 Table Non-targeted LC/MS-based metabolomics for association with CHD in ULSAM and validation in TwinGene.
(XLSX)

S3 Table Association between metabolites replicated in the univariable analysis and CHD in ULSAM and TwinGene, adjusted for established risk factors.
(XLSX)

S4 Table Association between LysoPCs and LysoPCs ratios and CHD in ULSAM, TwinGene and meta-analysis, adjusted for established risk factors.
(XLSX)

S5 Table Reclassification table in TwinGene when four metabolites are added to established risk factors.
(XLSX)

S6 Table Comparison of the association between MG 18∶2 and/or triglycerides with CHD in TwinGene.
(XLSX)

S7 Table Association between four metabolites and CHD in ULSAM when the follow-up is extended to 20-years, adjusted for established risk factors.
(XLSX)

S8 Table Association between four metabolites and CHD in ULSAM and TwinGene, adjusted for established risk factors and additional covariates.
(XLSX)

S1 Text Description of the included studies, protocol to perform metabolomics profiling and data processing, genotyping procedures and additional statistical methods.
(DOCX)

## References

[1] Suhre K, Shin SY, Petersen AK, Mohney RP, Meredith D, et al (2011) Human metabolic individuality in biomedical and pharmaceutical research. Nature 477: 54–60. 21886157 10.1038/nature10354 PMC3832838

[2] Magnusson M, Lewis GD, Ericson U, Orho-Melander M, Hedblad B, et al (2013) A diabetes-predictive amino acid score and future cardiovascular disease. Eur Heart J 34: 1982–1989. 23242195 10.1093/eurheartj/ehs424 PMC3703309

[3] Shah SH, Bain JR, Muehlbauer MJ, Stevens RD, Crosslin DR, et al (2010) Association of a peripheral blood metabolic profile with coronary artery disease and risk of subsequent cardiovascular events. Circ Cardiovasc Genet 3: 207–214. 20173117 10.1161/CIRCGENETICS.109.852814

[4] Boyanovsky BB, Webb NR (2009) Biology of secretory phospholipase A2. Cardiovasc Drugs Ther 23: 61–72. 18853244 10.1007/s10557-008-6134-7 PMC7101564

[5] Wilson PW, D'Agostino RB, Levy D, Belanger AM, Silbershatz H, et al (1998) Prediction of coronary heart disease using risk factor categories. Circulation 97: 1837–1847. 9603539 10.1161/01.cir.97.18.1837

[6] Lemaitre RN, Tanaka T, Tang W, Manichaikul A, Foy M, et al (2011) Genetic loci associated with plasma phospholipid n-3 fatty acids: a meta-analysis of genome-wide association studies from the CHARGE Consortium. PLoS Genet 7: e1002193. 21829377 10.1371/journal.pgen.1002193 PMC3145614

[7] Schunkert H, Konig IR, Kathiresan S, Reilly MP, Assimes TL, et al (2011) Large-scale association analysis identifies 13 new susceptibility loci for coronary artery disease. Nat Genet 43: 333–338. 21378990 10.1038/ng.784 PMC3119261

[8] Demirkan A, van Duijn CM, Ugocsai P, Isaacs A, Pramstaller PP, et al (2012) Genome-wide association study identifies novel loci associated with circulating phospho- and sphingolipid concentrations. PLoS Genet 8: e1002490. 22359512 10.1371/journal.pgen.1002490 PMC3280968

[9] CARDIoGRAMplusC4D Consortium (2013) Deloukas P, Kanoni S, Willenborg C, Farrall M, et al (2013) Large-scale association analysis identifies new risk loci for coronary artery disease. Nat Genet 45: 25–33. 23202125 10.1038/ng.2480 PMC3679547

[10] Miller M, Stone NJ, Ballantyne C, Bittner V, Criqui MH, et al (2011) Triglycerides and cardiovascular disease: a scientific statement from the American Heart Association. Circulation 123: 2292–2333. 21502576 10.1161/CIR.0b013e3182160726

[11] Do R, Willer CJ, Schmidt EM, Sengupta S, Gao C, et al (2013) Common variants associated with plasma triglycerides and risk for coronary artery disease. Nat Genet 45: 1345–1352. 24097064 10.1038/ng.2795 PMC3904346

[12] Rozenberg O, Shih DM, Aviram M (2003) Human serum paraoxonase 1 decreases macrophage cholesterol biosynthesis: possible role for its phospholipase-A2-like activity and lysophosphatidylcholine formation. Arterioscler Thromb Vasc Biol 23: 461–467. 12615663 10.1161/01.ATV.0000060462.35946.B3

[13] Gauster M, Rechberger G, Sovic A, Horl G, Steyrer E, et al (2005) Endothelial lipase releases saturated and unsaturated fatty acids of high density lipoprotein phosphatidylcholine. J Lipid Res 46: 1517–1525. 15834125 10.1194/jlr.M500054-JLR200

[14] Sekas G, Patton GM, Lincoln EC, Robins SJ (1985) Origin of plasma lysophosphatidylcholine: evidence for direct hepatic secretion in the rat. J Lab Clin Med 105: 190–194. 3973457

[15] Shamburek RD, Zech LA, Cooper PS, Vandenbroek JM, Schwartz CC (1996) Disappearance of two major phosphatidylcholines from plasma is predominantly via LCAT and hepatic lipase. Am J Physiol 271: E1073–1082. 8997228 10.1152/ajpendo.1996.271.6.E1073

[16] Schmitz G, Ruebsaamen K (2010) Metabolism and atherogenic disease association of lysophosphatidylcholine. Atherosclerosis 208: 10–18. 19570538 10.1016/j.atherosclerosis.2009.05.029

[17] Wang-Sattler R, Yu Z, Herder C, Messias AC, Floegel A, et al (2012) Novel biomarkers for pre-diabetes identified by metabolomics. Mol Syst Biol 8: 615. 23010998 10.1038/msb.2012.43 PMC3472689

[18] Fernandez C, Sandin M, Sampaio JL, Almgren P, Narkiewicz K, et al (2013) Plasma lipid composition and risk of developing cardiovascular disease. PLoS One 8: e71846. 23967253 10.1371/journal.pone.0071846 PMC3744469

[19] Stegemann C, Pechlaner R, Willeit P, Langley S, Mangino M, et al (2014) Lipidomics Profiling and Risk of Cardiovascular Disease in the Prospective Population-Based Bruneck Study. Circulation 10.1161/CIRCULATIONAHA.113.002500 24622385

[20] Tzoulaki I, Ebbels TM, Valdes A, Elliott P, Ioannidis JP (2014) Design and analysis of metabolomics studies in epidemiologic research: a primer on -omic technologies. Am J Epidemiol 180: 129–139. 24966222 10.1093/aje/kwu143

[21] Ganna A, Fall T, Lee W, Broeckling CD, Kumar J, et al (2014) A workflow for UPLC-MS non-targeted metabolomic profiling in large human population-based studies. bioRxiv

[22] Ganna A, Lee D, Ingelsson E, Pawitan Y (2014) Rediscovery rate estimation for assessing the validation of significant findings in high-throughput studies. Brief Bioinform 10.1093/bib/bbu033 25256289

[23] Magnusson PK, Almqvist C, Rahman I, Ganna A, Viktorin A, et al (2013) The Swedish Twin Registry: establishment of a biobank and other recent developments. Twin Res Hum Genet 16: 317–329. 23137839 10.1017/thg.2012.104

[24] Ganna A, Reilly M, de Faire U, Pedersen N, Magnusson P, et al (2012) Risk prediction measures for case-cohort and nested case-control designs: an application to cardiovascular disease. Am J Epidemiol 175: 715–724. 22396388 10.1093/aje/kwr374 PMC3324433

[25] Byberg L, Siegbahn A, Berglund L, McKeigue P, Reneland R, et al (1998) Plasminogen activator inhibitor-1 activity is independently related to both insulin sensitivity and serum triglycerides in 70-year-old men. Arterioscler Thromb Vasc Biol 18: 258–264. 9484991 10.1161/01.atv.18.2.258

[26] Lind L, Fors N, Hall J, Marttala K, Stenborg A (2005) A comparison of three different methods to evaluate endothelium-dependent vasodilation in the elderly: the Prospective Investigation of the Vasculature in Uppsala Seniors (PIVUS) study. Arterioscler Thromb Vasc Biol 25: 2368–2375. 16141402 10.1161/01.ATV.0000184769.22061.da

[27] Broeckling CD, Heuberger AL, Prenni JE (2013) Large scale non-targeted metabolomic profiling of serum by ultra performance liquid chromatography-mass spectrometry (UPLC-MS). J Vis Exp e50242. 23524330 10.3791/50242 PMC3639512

[28] Lind L, Fors N, Hall J, Marttala K, Stenborg A (2006) A comparison of three different methods to determine arterial compliance in the elderly: the Prospective Investigation of the Vasculature in Uppsala Seniors (PIVUS) study. J Hypertens 24: 1075–1082. 16685207 10.1097/01.hjh.0000226197.67052.89

[29] Lind L, Siegbahn A, Hulthe J, Elmgren A (2008) C-reactive protein and e-selectin levels are related to vasodilation in resistance, but not conductance arteries in the elderly: the prospective investigation of the Vasculature in Uppsala Seniors (PIVUS) study. Atherosclerosis 199: 129–137. 17991470 10.1016/j.atherosclerosis.2007.09.038

[30] Lind L, Siegbahn A, Ingelsson E, Sundstrom J, Arnlov J (2011) A detailed cardiovascular characterization of obesity without the metabolic syndrome. Arterioscler Thromb Vasc Biol 31: e27–34. 21546604 10.1161/ATVBAHA.110.221572

[31] Smith CA, Want EJ, O'Maille G, Abagyan R, Siuzdak G (2006) XCMS: processing mass spectrometry data for metabolite profiling using nonlinear peak alignment, matching, and identification. Anal Chem 78: 779–787. 16448051 10.1021/ac051437y

[32] Sumner LW, Amberg A, Barrett D, Beale MH, Beger R, et al (2007) Proposed minimum reporting standards for chemical analysis Chemical Analysis Working Group (CAWG) Metabolomics Standards Initiative (MSI). Metabolomics 3: 211–221. 24039616 10.1007/s11306-007-0082-2 PMC3772505

[33] Leening MJ, Vedder MM, Witteman JC, Pencina MJ, Steyerberg EW (2014) Net reclassification improvement: computation, interpretation, and controversies: a literature review and clinician's guide. Ann Intern Med 160: 122–131. 24592497 10.7326/M13-1522

