# Supplementary figures and images for: Large-scale Metabolomic Profiling Identifies Novel Biomarkers for Incident Coronary Heart Disease
Source: PLoS Genet. 2014 Dec 11;10(12):e1004801. doi: 10.1371/journal.pgen.1004801 (PMC4263376; doi:10.1371/journal.pgen.1004801)

A.

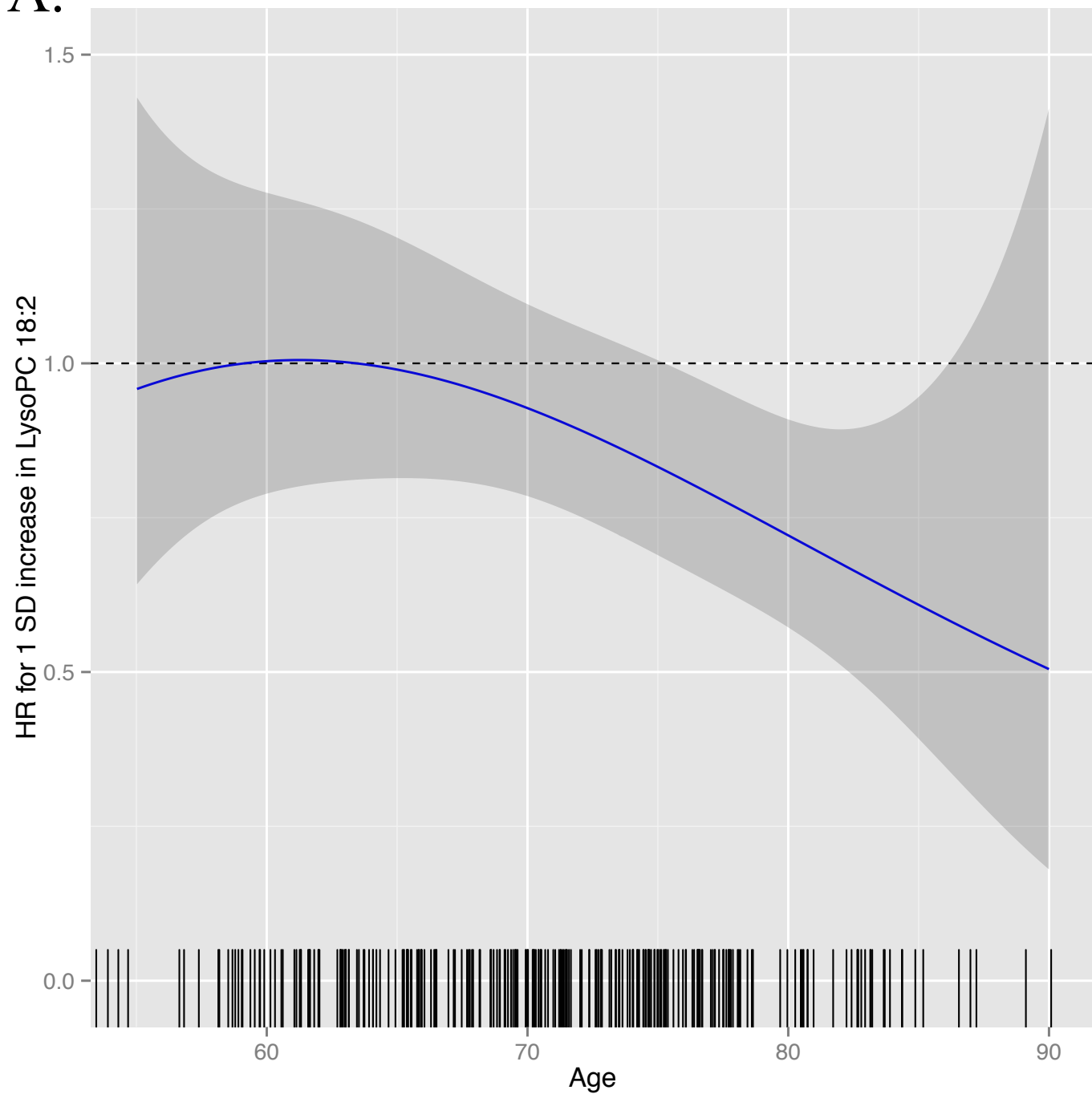

B.

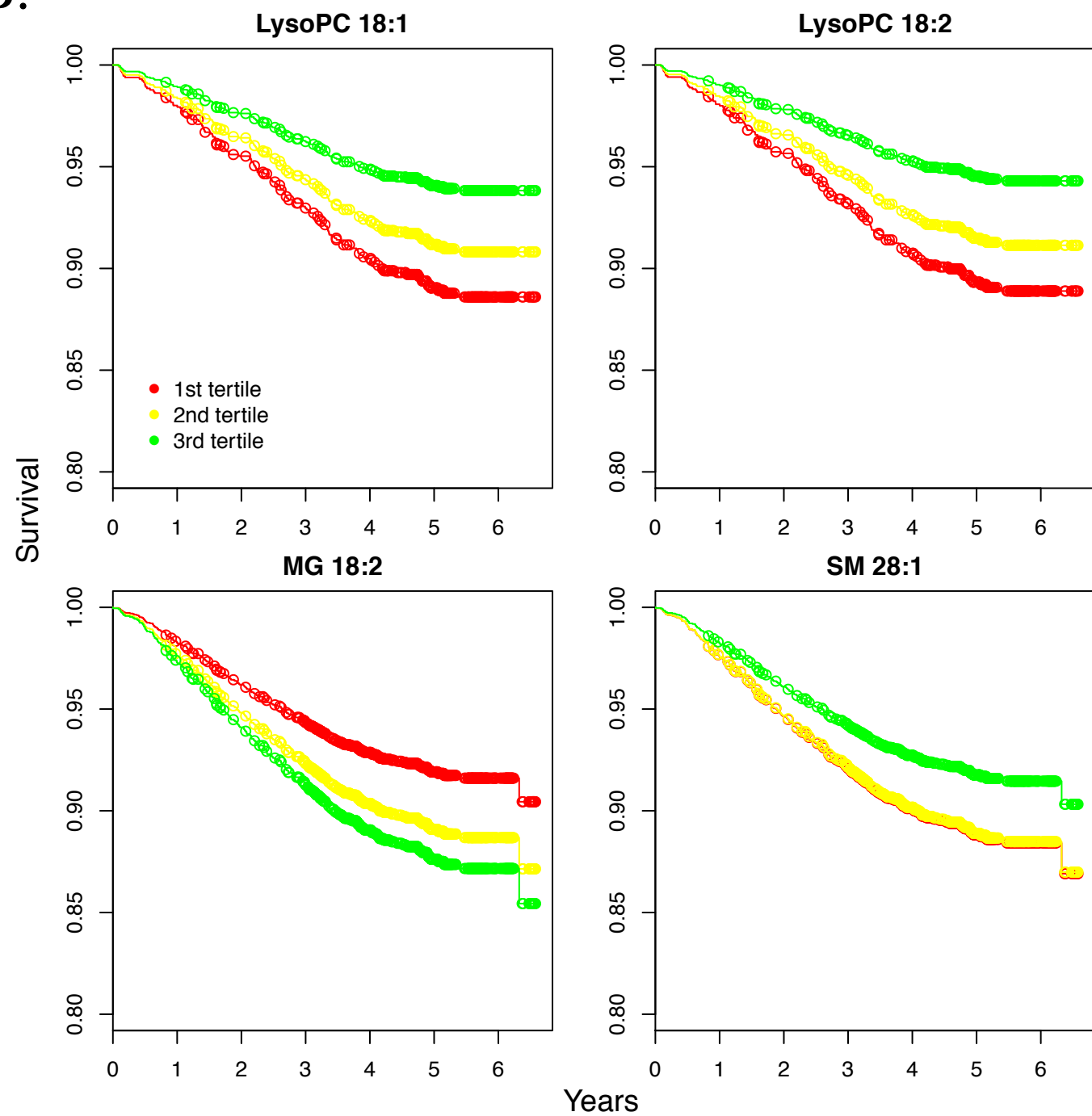

Supplement: S1 Figure — Panel A: Hazard Ratio (HR) for association between LysoPC 18∶2 and incident CHD as function of age, modelled using splines. The association between LysoPC 18∶2 and CHD is stronger at older age, starting from around 70-years old. Panel B: Survival curves for time-to-CHD for tertiles of each metabolite. We fixed the other covariates so that the curves are representative of a men, 77 years old, smoker, not antihypertensive drugs user and not diabetic with systolic blood pressure = 150, BMI = 26, LDL-C = 2.6 mmol/l, HDL-C = 1.3 mmol/l and triglycerides = 1.7 mmol/l. (PDF) [file pgen.1004801.s001.pdf]

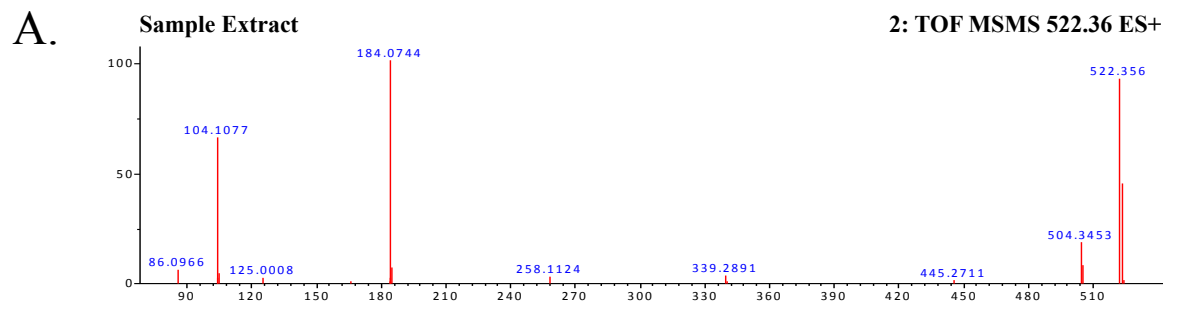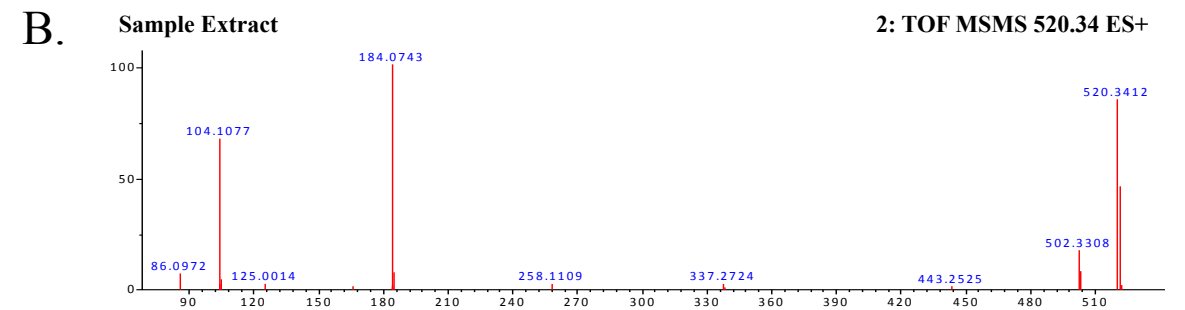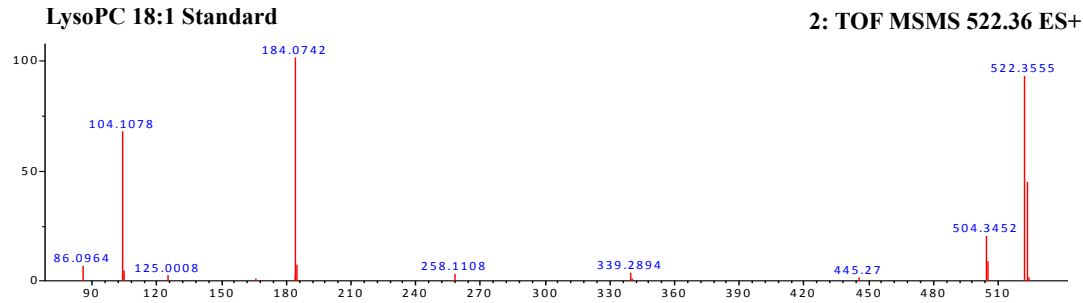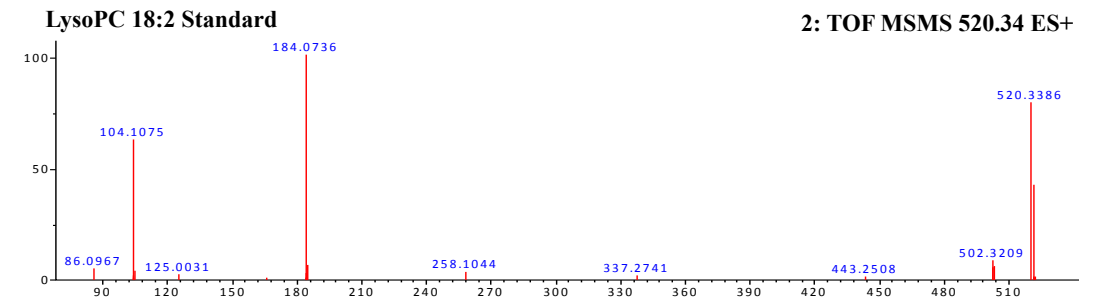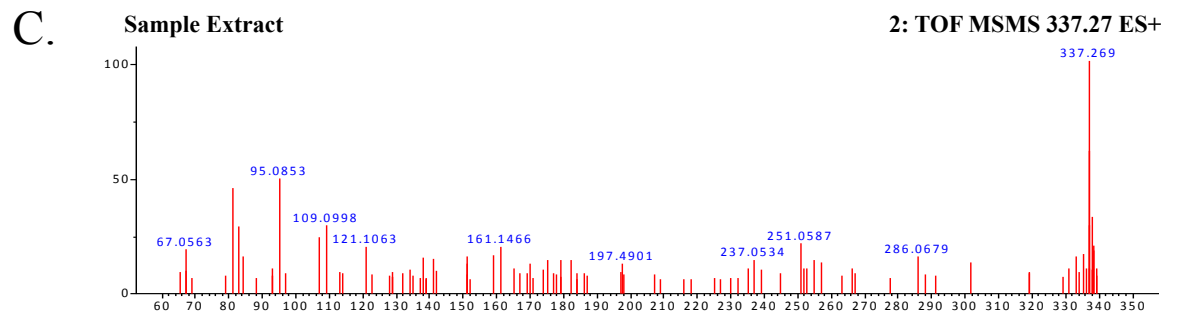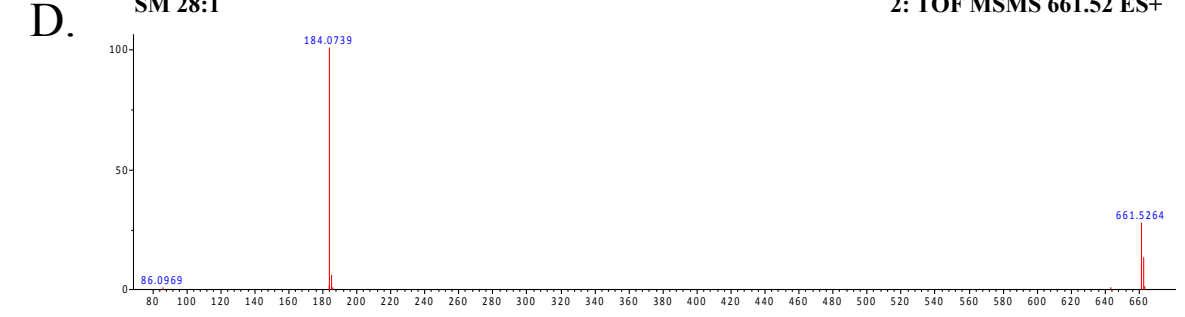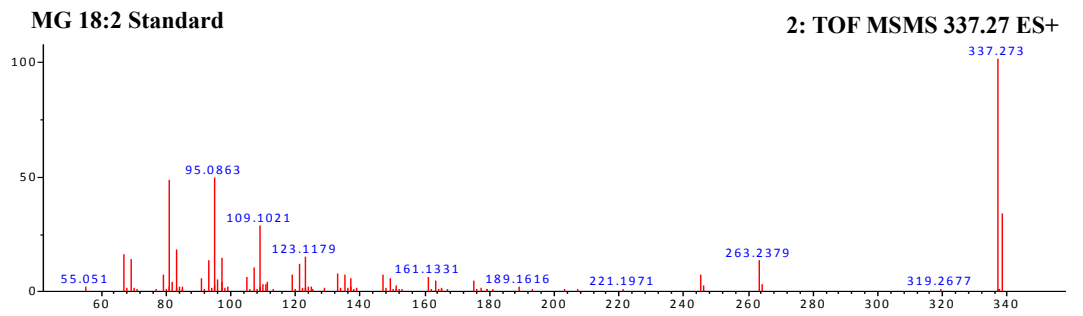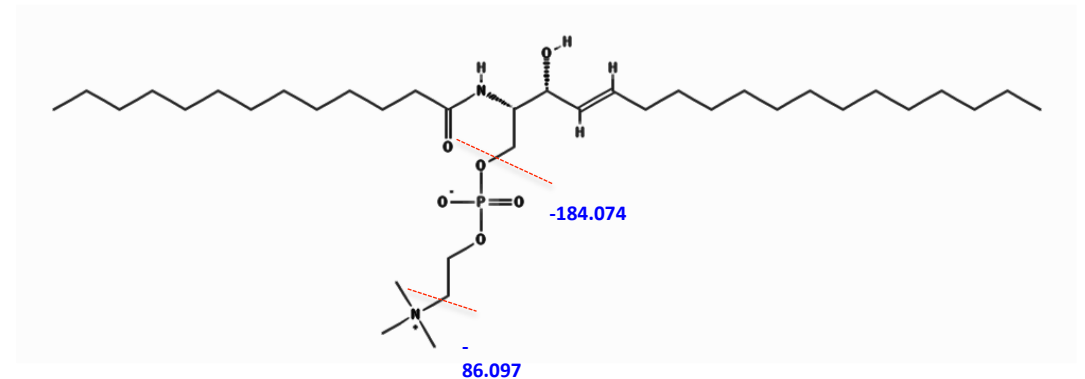

Supplement: S2 Figure — Product ion spectra of selected metabolites (upper spectrum) and their corresponding standards (lower spectrum) using a UPLC-QTOFMS operated in ESI positive mode. Panel A: Metabolite eluting at 6.37 minutes represented by [M+H]+ = 522.356 identified as LysoPC 18∶1. Panel B: Metabolite eluting at 5.78 represented by [M+H]+ = 520.341 identified as LysoPC 18∶2. Panel C: Metabolite eluting at 6.40 represented by [M+H]+ = 337.269 identified as MG 18∶2. Panel D: Product ion spectra of a SM 28∶1 (C36H73N2O6P, M 660.521 Da) and its fragmentation pattern using a UPLC-QTOFMS operated in ESI positive mode where the protonated molecule [M+H]+ = 661.526 and the fragment ions at m/z 184.074 and m/z 86.097 represented by the loss of a phosphocholine group and a choline group, respectively. (PDF) [file pgen.1004801.s002.pdf]

rs75729820

Plotted SNPs

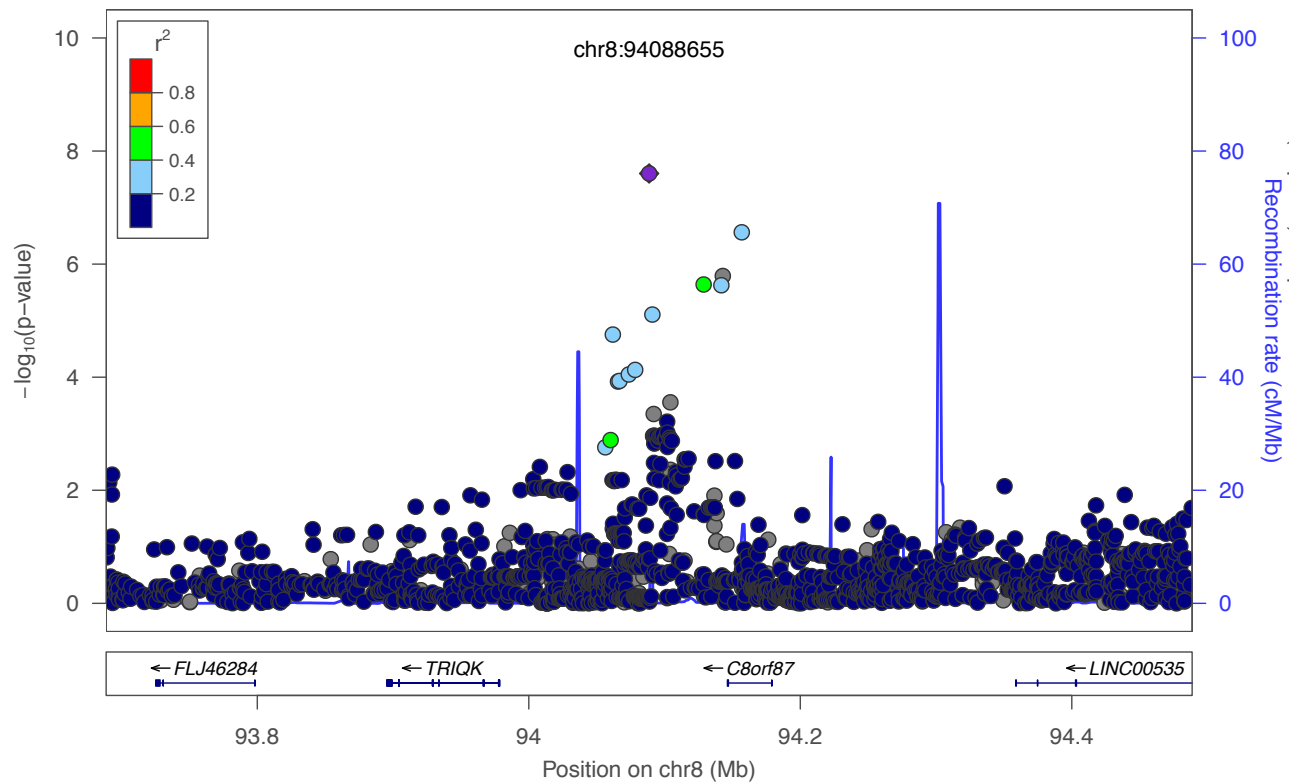

rs8141918

Plotted SNPs

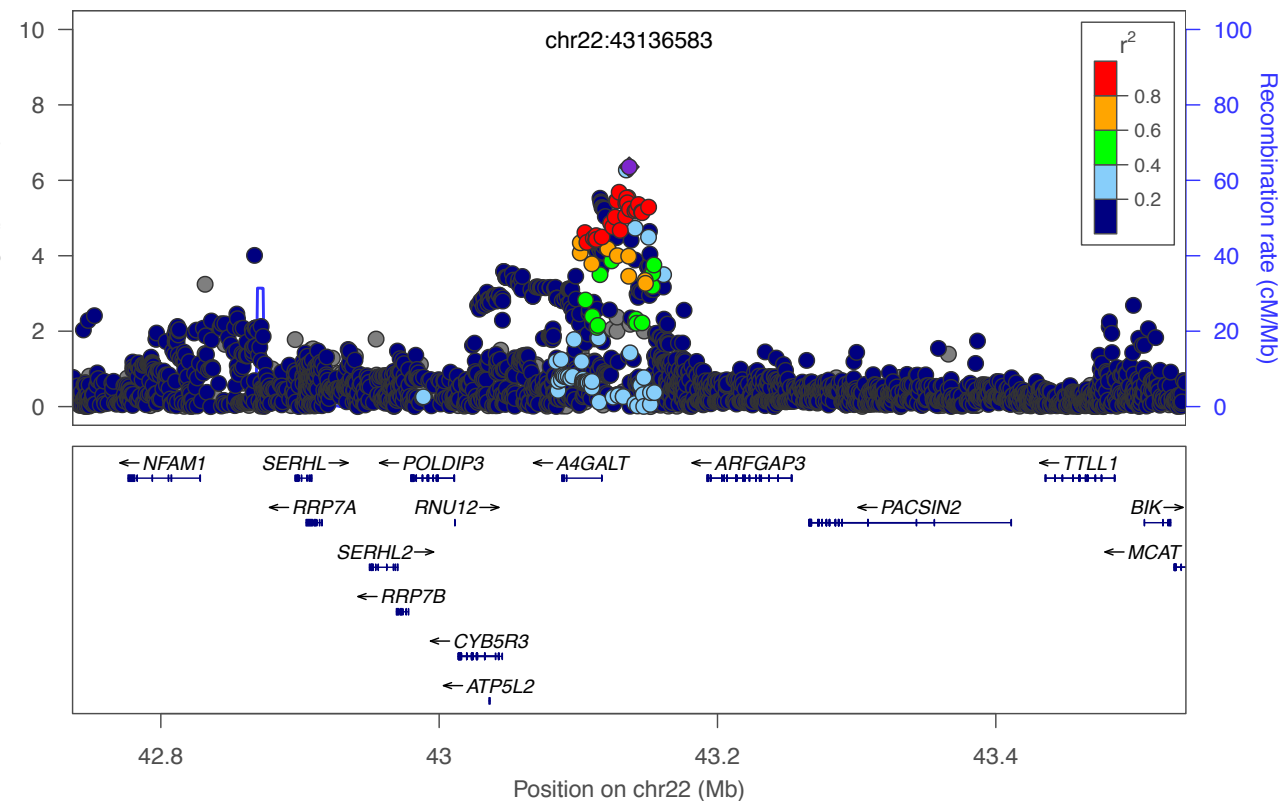

Supplement: S3 Figure — LocusZoom plots of the two top signals for association with LysoPC 18∶1. (PDF) [file pgen.1004801.s003.pdf]

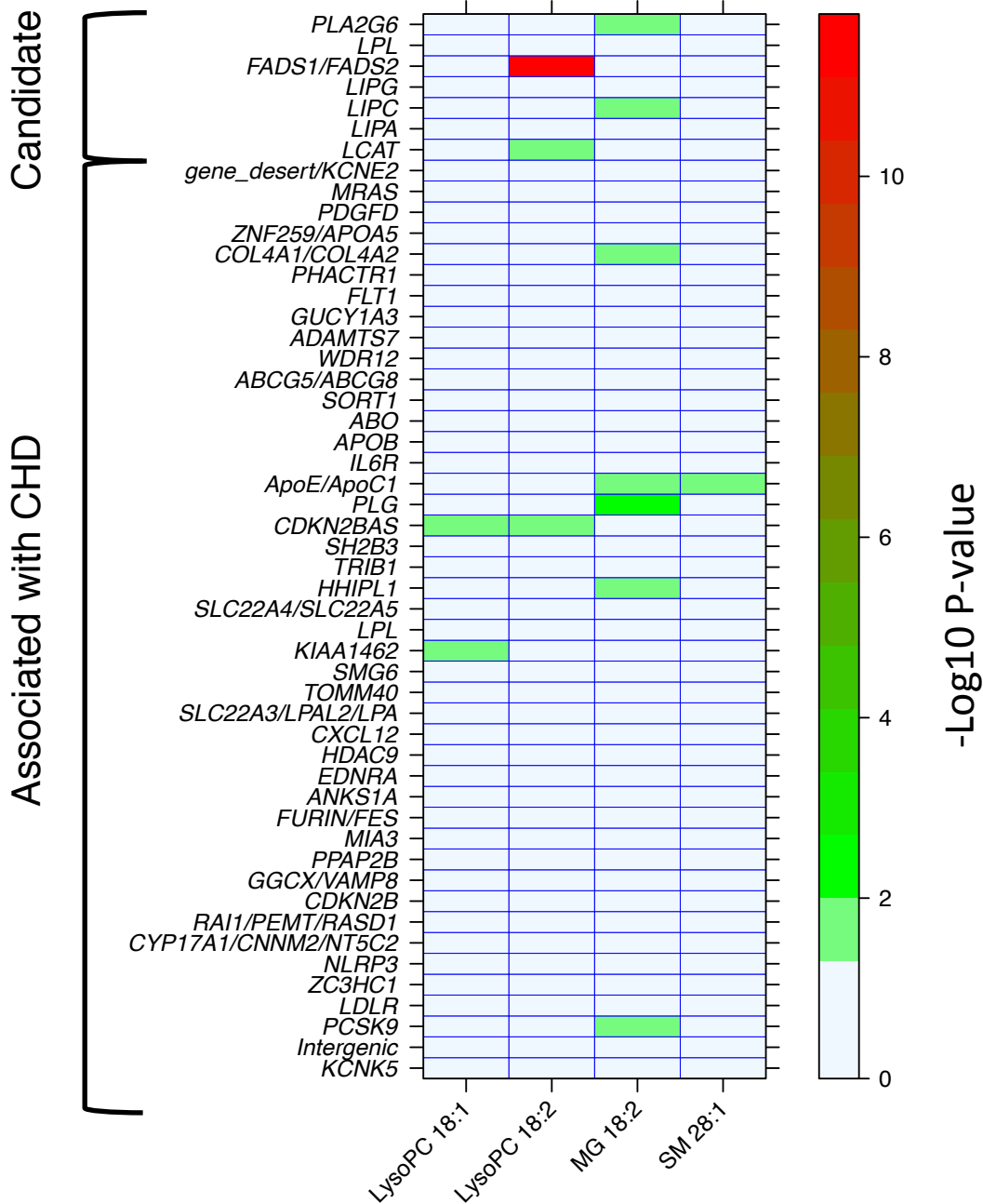

Supplement: S4 Figure — Minus log10(P-value) for association between four metabolites and 51 SNPs previously reported for association with CHD (44 SNPs) or selected from candidate pathways (7 SNPs) after adjustment for main cardiovascular risk factors. (PDF) [file pgen.1004801.s004.pdf]
