# Supplementary material for: Large-scale Metabolomic Profiling Identifies Novel Biomarkers for Incident Coronary Heart Disease
Source: PLoS Genet. 2014 Dec 11;10(12):e1004801. doi: 10.1371/journal.pgen.1004801 (PMC4263376; doi:10.1371/journal.pgen.1004801)
Supplement: S1 Text — Description of the included studies, protocol to perform metabolomics profiling and data processing, genotyping procedures and additional statistical methods. (DOCX) [file pgen.1004801.s013.docx]

**TEXT S1**

**Table of contents:**

**Expanded Methods and Results**

- **Page 2:** Cohort descriptions
- **Page 3:** Outcome assessment
- **Page 4:** Detailed description of laboratory procedures for UPLC-MS
- **Page 5:** Metabolomics analysis
- **Page 7:** Genotyping procedures
- **Page 8:** Candidate SNP selection
- **Page 9:** Additional statistical methods

References (**Page 10)**

**Cohort descriptions**

**TwinGene**

The Swedish Twin Registry is a population-based national register including over 194,000 Swedish twins born from 1886 to 2008 [23]. TwinGene is a longitudinal study nested within the Swedish Twin Register initiated to examine associations between genetic factors and cardiovascular disease in Swedish twins. Twins born before 1958 and who has participated in a telephone screening between 1998 and 2002 were re-contacted between April 2004 and December 2008. Health and medication data were collected from self-reported questionnaires, and a blood sampling kit was mailed to the subject who then contacted a local health care center for blood sampling and a health check-up. In total, 12,591 individuals (55% women) participated by donating blood to the study, and by answering questionnaires about life style and health.

In TwinGene, we utilized a case-cohort design by selecting all the incident cases of coronary heart disease, type 2 diabetes, ischemic strokes and dementia up to 31^st^ December 2010 and a sub-cohort (controls) of 1,643 individuals (43% women). Since it has been previously shown to improve the study efficiency [[2](#_ENREF_2)4], the subcohort was stratified on median age and sex, and for each of the four strata, we randomly selected a number of participants proportional to the corresponding number of cases. Fasting blood samples were collected at the local healthcare facility and sent with overnight mail to the Karolinska Biobank where they were frozen at -80° C until analysis. In the present analysis, we included individuals from the sub-cohort and incident cases of coronary heart diseases and excluded non-fasting individuals (N=32) and participants with previous coronary heart disease (CHD) events (N=136). The final number of individuals included in the analysis was 1,670.

**ULSAM**

Men born between 1920 and 1924 in Uppsala, Sweden were invited to participate at age 50 (N=2,841) in this longitudinal cohort study, which was started in 1970 [[25](#_ENREF_3)]; 81.7% (N=2,322) participated. Subjects were reinvestigated at the ages of 60, 70, 77, 82 and 88 years. Information collected includes a medical questionnaire, blood pressure and anthropometric measurements, glucose tolerance test and 24-hour ambulatory blood pressure. Blood samples for metabolomics profiling and assessment of established cardiovascular risk factors were frozen immediately after separation of plasma and stored at -80° C until analysis. Plasma samples were available for 1,138 participants at 70 years of age (1991-1994). We excluded 110 participants that experienced a CHD event before baseline, resulting in 1,028 individuals included in the current study.

**PIVUS**

Prospective Investigation of the Vasculature in Uppsala Seniors (PIVUS) (http://www.medsci.uu.se/pivus/pivus.htm) is a community-based study where all men and women at age 70 living in Uppsala, Sweden were invited to participate in 2001. The 1,016 participants (50% women) have been extensively phenotyped, as described previously [[26](#_ENREF_4)], and on the Internet (www.medsci.uu.se/pivus/). Blood samples were frozen immediately after separation of plasma and stored at -80° C until analysis. Metabolomic profiling was performed on serum samples from 970 fasting participants.

**Outcome assessment**

Information regarding incident CHD was collected from the Swedish National In-Patient Register and the Cause of Death Register, and was defined as hospitalization or death with any of the following primary diagnoses: acute myocardial infarction and unstable angina (ICD-10: I20.0, I21, I22; ICD-9: 410, 411B; ICD-8: 410, 411 and surgical codes: FNG02, FNG05, FNC, FND, FNE). We recorded the follow-up time from the baseline examination to the date of the first CHD event (as defined above), emigration from Sweden, death or end of follow-up (Dec 31, 2010), whichever occurred first.

**Detailed description of laboratory procedures for UPLC-MS**

**Sample preparation**

Serum samples were thawed and 100 µL of serum was transferred to 400 µL methanol in 96-well format to precipitate proteins. This 80% methanol solution was stored at -20°C overnight, and then centrifuged for 30 minutes at 3800g in 4°C to pellet precipitated protein. The supernatant was aliquoted to three separate 96-well plates, sealed using a heat-seal foil, and stored at -20°C until analysis. Plate analysis order was completely randomized for each set of injections, and plates were run in batches of two plates, reflecting the autosampler capacity. Within each set of two plates, the run order was again completely randomized to prevent injection order artifacts. Duplicate injections were performed for all samples, with the second set of injections performed upon completion of the first set of injections for all samples.

**UPLC-MS data acquisition**

Previous to each batch of two plates of samples, instrument maintenance (cone cleaning, mass calibration, and detector gain calibration) was performed, and a quality control standard mix was injected. Two conditioning and three QC injections were performed, with 1 μL injections of a 20% methanol solution containing 2 μg/mL each of caffeine, terfenadine, sulfadimethoxime, and reserpine. The QC standards were evaluated for retention time (+/- 0.05 minutes), signal intensity (<25% relative standard deviation), and mass accuracy (< 3 ppm). This approach is designed to prevent acquisition of low-quality data. QC steps post-acquisition described in the body of the manuscript were then used to remove poor injections/samples.

One μL injections of protein-precipitated serum were performed on a Waters Acquity UPLC system. Separation was performed using a Waters Acquity UPLC C8 column (1.8 µM, 1.0 x 100 mm), using a gradient from solvent A (95% water, 5% methanol, 0.1% formic acid) to solvent B (95% methanol, 5% water, 0.1% formic acid). Injections were made in 100% A, which was held for 0.1 min, ramped to 40% B in 0.9 minutes, to 70% B over two minutes, and to 100% B over 8 minutes. The mobile phase was held at 100% B for 6 minutes, returned to starting conditions over 0.1 minutes, and allowed to re-equilibrate at for 5.9 minutes. Flow rate was constant at 140 µL/min for the duration of the run. The column was held at 50°C, while samples were held at 10°C.

Column eluent was infused into a Waters Xevo G2 TOF MS fitted with an electrospray source. Data was collected in positive ion mode, scanning from 50-1200 at a rate of 5 scans per second. Scans were collected alternatively in MS mode at collision energy of 6 V and in idMS/MS mode using higher collision energy (15–30 V). IdMS/MS (also called MS^E^) allows for unbiased view of MS/MS fragmentation without additional experiments [[34](#_ENREF_5)].

Calibration was performed prior to sample analysis via infusion of sodium formate solution, with mass accuracy within 1 ppm. The capillary voltage was held at 2200V, the source temp at 150°C, and the desolvation temperature at 350°C at a nitrogen desolvation gas flow rate of 800 L/hr. The quadrupole was held at collision energy of 6 volts. Raw data files were converted to .cdf format using Waters DataBridge software for processing.

**Metabolomics analysis**

Our metabolomics pipeline has been previously described[21] and the code to process the data is made publically available (<https://github.com/andgan/metabolomics_pipeline>).

**Feature detection using XCMS**

The first step in the metabolomics workflow is the detection, alignment, grouping and imputation of the metabolic features. This is done by the XCSM software implemented in R. Below are the parameters we used:

- Peak detection (xcmsSet *function*): *method="centWave", ppm=25, peakwidth=c(2:15), snthresh=8, mzCenterFun="wMean", integrate=2, mzdiff=0.05 prefilter=c(1,5);*
- Peak alignment (*rector* function): *method="obiwarp",plottype="deviation";*
- Peak grouping (*group* function): *bw=2, minfrac=0.05, max=100, mzwid=0.01*
- Peak filling (*fillPeaks.chrom* function).

All the other parameters were set to default.

We detected 9,755, 10,162 and 7,522 metabolic features in TwinGene, ULSAM and PIVUS, respectively. Each feature is characterized by a specific mass-to-charge ratio (m/z) and retention time. A single metabolite is normally represented by more than one feature. We identified common features between the three studies by matching on mass-to-charge ratio and retention time followed by manual inspection of the spectra.

**Parameter selection**

The parameters used in XCMS to detect, align and group peaks can drastically change the number and quality of the identified features. The authors of XCMS have suggested parameter values for different UPLC/MS instruments; both in a published paper [[35](#_ENREF_7)] and in the online version of XCMS. We randomly selected a small number of samples (n=20-40) with duplicate or triplicate injections and tried several parameter configurations within a reasonable interval around the suggested values. We selected the parameter configuration that maximized the intra-replicates correlation. Moreover, we performed manual inspection of the plots generated by the peak detection and grouping algorithms to evaluate whether the selected configuration was appropriate.

**Log-transformation and normalization**

Metabolic features intensities were log-transformed and normalized to take into account factors of unwanted variation. We performed ANOVA-type normalization approach which has been shown to increase correlation between duplicates compared to other normalization methods in our data [[21](#_ENREF_6)]. Specifically, we fitted a linear regression for association between each feature intensity and the factors of unwanted variability. We then used the residuals from the regression as new feature intensities. The factors of unwanted variability can be identified by studying the association between several technical variables and the first principal components. In each study we adjusted for the following factors:

- TwinGene: retention time correction, analysis date, storage time, unknown cluster effect.
- ULSAM: retention time correction, analysis date, sample collection, plate effect
- PIVUS: retention time correction, analysis date, storage time, season effect

*Exclusion of outliers and uncorrelated features*

We manually excluded samples with abnormal total feature intensity. We also excluded those features with low Spearman correlation between duplicates. The average correlation between duplicated features was 0.38 in TwinGene, 0.46 in ULSAM and 0.43 in PIVUS.

**Metabolite detection**

Results from the association analysis are normally characterized by clusters of highly correlated features. Each cluster includes features with very similar retention time, but different masses, due the inherent fragmentation pattern of each metabolite. We combined features with strong correlation and similar retention time to reconstruct the fragmentation spectra [21] and used the spectra for annotation.

There are four annotation approaches, each with a different confidence level:

- The first approach has the highest confidence (level 1 according to the levels proposed by the Metabolomics Standard Initiative [MSI][[32](#_ENREF_8)]) and it is based on matching accurate mass, fragmentation pattern, and retention time with the in-house spectral library of authentic standards collected under the same experimental conditions.
- The second approach (MSI level 2) is based on spectrum and/or m/z similarities, but not retention time similarity, and their annotation relies on information available in public databases.
- The third approach (MSI level 3) uses a combination of spectral data, accurate mass, and retention time to assign the metabolite to a chemical class (without knowing the exact origin of the metabolite).
- Finally, if all the other approaches have failed in the annotation of the metabolite or metabolite class, the metabolite is annotated as “unknown” (MSI level 4).

**Genotyping procedures**

TwinGene and PIVUS participants were genotyped with Illumina Human OmniExpress (≈700,000 SNPs), while ULSAM participants were genotyped with Illumina Human Omni2.5M (≈2,500,000 SNPs).

All three studies underwent the same quality control and imputation procedures. Specifically, we used these inclusion criteria:

- Minor allele frequency greater than 3%.
- Hardy-Weinberg Equilibrium test P-value greater than 1*10^-6^.
- Genotype rate per SNP greater than 95%.
- Genotype rate per individual greater than 97%.
- Reported sex equal to sex identified using the X chromosome.
- Info score > 0.4.

SNPs that were not directly genotyped were imputed using IMPUTE2 (<http://mathgen.stats.ox.ac.uk/impute/impute_v2.html>) and 1000 Genome CEU Phase I version 3 as reference panel.

We performed a fixed effect meta-analysis with genomic control to combine the results from the three studies. We excluded SNPs with minor allele frequency <5% and those detected in only one study after meta-analysis before any further analyses.

**Candidate SNPs selection**

We selected 51 candidate SNPs and studied the association with four metabolites associated with incident CHD. Forty-four of these SNPs were selected because reported for association with CHD by the CARDIOoGRAMplusC4D consortia [[9](#_ENREF_9)]. The remaining 7 SNPs were chosen from genes involved in biological pathways related to the studied metabolites. For each gene, we selected the SNP that was most commonly reported by GWAS studies close to the candidate gene irrespectively of the investigated phenotype.

**Additional statistical methods**

**Prediction measures**

In TwinGene, we calculated the individual 10-year risk of experiencing a CHD event to determine discrimination (C-index) and the Net Reclassification Index (NRI). To calculate the NRI, we selected cut-offs of 10% and 20%, often used thresholds in previous literature[33]. Since the maximum follow-up time in TwinGene was 6.6 years, we predicted the 10-year survival by fitting smooth splines to the baseline survival. The prediction measures were weighted by inverse of sampling probability and confidence intervals were calculated via bootstrapping. The base model included the same risk factors included in the Framingham risk equation: sex, age, systolic blood pressure, current smoking, antihypertensive treatment, total cholesterol, high-density lipoprotein cholesterol [HDL-C] and prevalent diabetes.

**Mendelian randomization analysis**

In the Mendelian randomization analysis, we constructed one instrumental variable (IV) for each metabolite as a weighted sum of independent SNPs associated with the metabolite in the GWAS meta-analysis with P-value < 5x10^-5^. To avoid the inclusion of pleiotropic SNPs, we excluded all SNPs correlated (r^2^>0.2 within 5Mb) with any SNP previously reported for association with cardiometabolic traits in the GWAS catalogue. In addition, we manually inspected the IV estimates for each SNP and excluded those SNPs with outlying estimates. The IV was tested for association with CHD using the results from the CARDIOoGRAMplusC4D consortium. To plot the results and perform the statistical testing, we used the *gtx* package implemented in R.

**False discovery rate in the validation population**

We recently introduced a measure called vFDR (FDR in the validation) that estimates the expected number of false positives in the validation study [22].

This quantity is formally defined as:

$$vFDR=E(\frac{V_{v}}{R_{v}} | R_{v}> 0)$$

where $R_{v}$is the number of declared significant results in the validation study and $V_{v}$ is the number of false positives among those.

These quantities are estimated using a *t*-mixture approach [36], meaning that the distribution of the effect sizes $\delta$ in the discovery study can be obtained from a mixture of central and noncentral *t*-distribution, each observed with a certain probability $\pi$. Importantly, we only used data from the discovery study (beta and standard errors) to determine these quantities and we assumed that the discovery and validation samples were homogeneous, sharing the same underlying effect sizes and proportion of truly nonsignificant features.

We estimated two mixture components with a proportion of true null hypotheses equal to 0.63. This resulted in a vFDR of 0.23%. Calculation can be obtained online from: <http://fafner.meb.ki.se/personal/yudpaw/rdr/> or using the R package ‘RDR’ available at <http://fafner.meb.ki.se/personal/yudpaw/?page_id=13>.

**REFERENCES**

34. Abdel-Ghany SE, Day I, Heuberger AL, Broeckling CD, Reddy AS (2013) Metabolic engineering of Arabidopsis for butanetriol production using bacterial genes. Metab Eng 20: 109-120.

35. Patti GJ, Tautenhahn R, Siuzdak G (2012) Meta-analysis of untargeted metabolomic data from multiple profiling experiments. Nat Protoc 7: 508-516.

36. Pawitan Y, Murthy KR, Michiels S, Ploner A (2005) Bias in the estimation of false discovery rate in microarray studies. Bioinformatics 21: 3865-3872.
